# Effect of synthetic hormones on reproduction in *Mastomys natalensis*

**DOI:** 10.1007/s10340-017-0894-4

**Published:** 2017-08-14

**Authors:** Apia W. Massawe, Rhodes H. Makundi, Zhibin Zhang, Ginethon Mhamphi, Ming Liu, Hong-Jun Li, Steven R. Belmain

**Affiliations:** 1 Pest Management Centre, Africa Centre of Excellence for Innovative Rodent Pest Management and Biosensor Technology Development, Sokoine University of Agriculture, Morogoro, Tanzania; 2 State Key Laboratory of Pest Management of Insects and Rodents, Institute of Zoology, Chinese Academy of Sciences, Beijing, 100101 China; 3 State Key Laboratory of Reproductive and Stem Cell Biology, Institute of Zoology, Chinese Academy of Sciences, Beijing, 100101 China; 4 Natural Resources Institute, University of Greenwich, Chatham Maritime, Kent, ME4 4TB UK

**Keywords:** Contraceptive bait, Fertility control, Levonorgestrel, Multimammate rat, Quinestrol

## Abstract

Rodent pest management traditionally relies on some form of lethal control. Developing effective fertility control for pest rodent species could be a major breakthrough particularly in the context of managing rodent population outbreaks. This laboratory-based study is the first to report on the effects of using fertility compounds on an outbreaking rodent pest species found throughout sub-Saharan Africa. *Mastomys natalensis* were fed bait containing the synthetic steroid hormones quinestrol and levonorgestrel, both singly and in combination, at three concentrations (10, 50, 100 ppm) for 7 days. Consumption of the bait and animal body mass was mostly the same between treatments when analysed by sex, day and treatment. However, a repeated measures ANOVA indicated that quinestrol and quinestrol + levonorgestrel treatments reduced consumption by up to 45%, particularly at the higher concentrations of 50 and 100 ppm. Although there was no clear concentration effect on animal body mass, quinestrol and quinestrol + levonorgestrel lowered body mass by up to 20% compared to the untreated and levonorgestrel treatments. Quinestrol and quinestrol + levonorgestrel reduced the weight of male rat testes, epididymis and seminal vesicles by 60–80%, and sperm concentration and motility were reduced by more than 95%. No weight changes were observed to uterine and ovarian tissue; however, high uterine oedema was observed among all female rats consuming treated bait at 8 and 40 days from trial start. Trials with mate pairing showed there were significant differences in the pregnancy rate with all treatments when compared to the untreated control group of rodents.

## Key message

- Contraceptive hormones quinestrol and levonorgestrel were able to limit the reproductive capacity of the most common rodent pest species found across sub-Saharan Africa, the multimammate rat *Mastomys natalensis.*
- Consumption rates of bait treated with the hormones were similar between males and females and to that observed in the untreated control, with no significant effects on body mass.
- Pregnancy rates were much lower among paired animals where one or both sexes consumed contraceptive hormones in comparison with untreated pairs.

## Introduction

Rodent population outbreaks are well known to occur in many parts of the world where such outbreaks are caused by a variety of abiotic, biotic and anthropogenic factors (Singleton et al. 2010). The multimammate rats, genus *Mastomys*, are indigenous to sub-Saharan Africa where population outbreaks have been documented since the early nineteenth century (Fiedler 1988; Leirs 1995). Rodent population outbreaks resulting in high agricultural losses in eastern and southern Africa are largely attributed to *Mastomys natalensis* (Leirs et al. 1994; Mwanjabe et al. 2002; Makundi and Massawe 2011). *M*. *natalensis* are considered a threat to many cereal crops (Mulungu et al. 2011) with economic losses especially high among small holder farmers (Stenseth et al. 2003; Makundi et al. 2005; Makundi and Massawe 2011). *M*. *natalensis* are also reservoirs of several zoonotic diseases of public health concern (Meerburg et al. 2009; Katakweba et al. 2012; Bordes et al. 2015; Morand et al. 2015). There are several abiotic and biotic explanatory factors associated with outbreaks of *M*. *natalensis* in Africa (Leirs 1992; Massawe et al. 2011). Outbreaks of *M*. *natalensis* seem to originate locally (Leirs et al. 1994) and are associated with rainfall patterns (Leirs et al. 1997). When environmental factors (e.g. prolonged rainfall, early onset rain, increased vegetation cover) are favourable, rapid reproduction occurs, enabling *M*. *natalensis* to build up in numbers within a relatively short time period (Leirs 1995).

Rodent management strategies in agro-ecological systems vary according to which rodent pest species are present, crop type, method of cropping, and the availability, affordability and cost–benefit of rodent management methods (Singleton and Petch 1994). Rodent control methods largely rely on the use of rodenticides (Buckle and Smith 2015). Both acute and chronic rodenticides have been used extensively during rodent outbreaks (Brown et al. 1997, 2002; Ngowo et al. 2005). Through increased rates of recruitment, rodent populations can rapidly recover after efforts to reduce populations through poisoning, trapping, hunting and other mortality-focussed population management strategies (Singleton et al. 2007). As described by Stenseth et al. (2001), the use of mortality control results in increased reproductive compensation and survival within short-lived, fast breeding animals such as rodents; thus populations rapidly recover. In contrast to mortality-based control options, the use of fertility control has been argued to be more ecologically sound (Stenseth et al. 2001) whereby infertile animals can remain in the population, therefore sustaining density dependant feedback to recruitment and survival (Zhang 2000). However, some compensation at the population level can still occur through higher survival of juveniles (Jacob et al. 2004; Williams et al. 2007). Managing populations through limiting fertility has also been argued to be more humane (Barlow 2000), and more safe and cost effective (Hone 1992) than the use of mortality control.

Fertility limiting compounds can cause permanent or temporary sterility in either sex, reduce the number of offspring or impair the fertility of offspring produced (Humphrys and Lapidge 2008) through a reduction in either fertility or fecundity (Bomford 1990). Several anti-fertility compounds have been used in controlling reproduction in various animal species, through contraception or sterilization (Kirkpatrick and Turner 1985; Tuyttens and Macdonald 1998; Fagerstone 2002; Jacob et al. 2008; Massei and Cowan 2014). The most common approach to wildlife contraception has been through the use of steroid hormones, particularly natural and synthetic oestrogens, progestins and androgens (Massei and Cowan 2014). Other chemical types such as alpha-chlorohydrin (sterilant) and bromocriptine (enzyme inhibitor of prolactin) have also been used for wildlife management (Sridhara and Dubey 2006). Synthetic progestins such as norgestomet, melengestrol acetate, megestrol acetate and levonorgestrel have been widely used in zoo animals, livestock and wildlife (Nave et al. 2002). In general, practical use of fertility control often faces problems with poor palatability and repeated baiting of contraceptive compounds.

Zhang (2015) demonstrated that quinestrol and levonorgestrel delivered at low dosage (10 ppm) could deliver long-term anti-fertility effects in several wild rodent species. Several studies have indicated that a single baiting of quinestrol and/or levonorgestrel at a dosage of 10–50 μg/ml (0.001–0.005%) delivered at the start of the rodent breeding season can successfully limit breeding for 1–2 years (Zhang 2015). Levonorgestrel is a progesterone analogue used as an emergency contraceptive to prevent pregnancy by preventing or interrupting ovulation and egg implantation in humans and other animals (Gemzell-Danielsson and Marions 2004; Novikova et al. 2007). It affects the cervical mucus or the ability of the sperm to bind to the egg (Asa and Porton 2005). It is still unclear whether levonorgestrel has effects on fertilization or implantation (Novikova et al. 2007). Quinestrol is a synthetic oestrogen homologue employed in many long-term oral contraceptives for human use (Zhao et al. 2007). Little is known regarding the effect of quinestrol on male fertility (Massei and Cowan 2014). Trials with quinestrol and levonorgestrel have shown some population limiting effects with rodent species such as Brandt’s voles (*Lasiopodomys brandtii*), Mongolian gerbils (*Meriones unguiculatus*) and plateau pikas (*Ochotona curzoniae*) (Zhao et al. 2007; Wang et al. 2011; Liu et al. 2012, 2016; Lv and Shi 2012; Qu et al. 2015). Anti-fertility effects of these compounds appear to impair reproductive performance of rodents by reducing the size and function of male reproductive organs, interfering with spermatogenesis, decreasing sperm concentration and motility and reducing female pregnancy rates and litter size through inducing uterine oedema (Wang et al. 2011; Lv and Shi 2012; Fu et al. 2013). Both compounds decompose quickly under field conditions, with half-lives of 5–16 days in soil and less than 3 days in water (Tang et al. 2012a, b; Zhang et al. 2014). Non-target effects on birds appear to be minimal (Qu et al. 2015).

The current study evaluates the potential effects of levonorgestrel and quinestrol on bait consumption and reproductive performance of *Mastomys natalensis* to determine whether the compounds are sufficiently palatable and have negative effects on reproductive potential of this rodent species. Our laboratory-based study focused on four aspects: (1) bait palatability; (2) effects on body and reproductive organ weight; (3) physiological changes in reproductive organs and cells; and (4) effects on birth rate and litter size.

## Materials and methods

### Experimental animals

The study was conducted in laboratories of the Pest Management Centre, Sokoine University of Agriculture, Morogoro, Tanzania (6°50′42.66″S, 37°39′29.14″E). Wild live captured adult *Mastomys natalensis* were used in the laboratory experiments. Animals were captured using Sherman LFA aluminium traps (H.B. Sherman Traps, Tallahassee, Florida) baited with peanut butter mixed with coarse maize flour in fallow agricultural fields owned by Sokoine University of Agriculture, which granted permission for the trials to take place. A total of 316 animals (158 males and 158 females) were captured between 25 September and 27 October 2015 and caged separately for at least two weeks to acclimatize to laboratory conditions (12:12 h light/dark, 24–30 °C, 40–60% rh [relative humidity]) before the baiting experiments were conducted. Captured animals were weighed and sexed, and animals of approximately the same weight (37 ± 5 g) were used for all trials, ensuring equal mean weights between sexes. All animals were fed ad libitum on standard pellet bait (see below) and water with wood shavings for nesting material. Female animals with a closed vagina at the time of capture were used for all trials.

### Bait preparation

Ten kilograms of maize flour was combined with 250 g of fish meal to give a 2.5% w/w fish meal in maize flour admix. The maize/fish meal flour was mixed with 20 l of boiling water and cooked for 15 min while being continuously stirred to form a stiff paste; the paste was left to cool to room temperature. The base rodent bait is then made by thoroughly mixing two-thirds roughly crushed maize (6.66 kg) and one-third of the maize/fish meal flour (3.33 kg). The bait is then passed through a mechanical pelletiser (NMG-744, Nikai Mfg., United Arab Emirates), to provide 10 kg of standard rodent bait.

In order to make contraceptive baits, powdered quinestrol and levonorgestrel (Beijing Zizhutiangong Science and Technology Ltd, China) were weighed in 0.1, 0.5 and 1 g quantities to prepare rodent bait at concentrations of 10, 50 and 100 ppm, respectively, in 10 kg of standard rodent bait. For the quinestrol + levonorgestrel combination, the compounds were mixed equally at a ratio of 1:1. Each quantity of contraceptive was dissolved in 100 ml of ethanol at 60–70 °C. The ethanol-contraceptive solution was then mixed with a sugar solution made from 200 g sucrose in 1000 ml water. This sugar-contraceptive solution was thoroughly mixed with the crushed maize just before adding the maize flour and fish meal paste. The plain bait was similarly prepared without contraceptives. All pelletized baits were dried in the shade at ambient temperature and stored in dark dry conditions until required.

### Bait acceptance and weight loss

Bait acceptance was evaluated using 50 males and 50 females adult *M*. *natalensis*. Each animal was kept in a separate animal cage and provided with 10 g of plain bait (control) or bait containing different concentrations of contraceptives (10, 50 and 100 ppm for each treatment of quinestrol, (QE), levonorgestrel (LNG) or quinestrol + levonorgestrel (QE + LNG), with five animals of each sex per treatment. Each animal was provided with fresh bait (10 g) every day for seven consecutive days. Water was supplied ad libitum for each animal. The body weight of each animal was recorded before feeding with the contraceptive or plain bait and thereafter daily for seven days. The amount of bait consumed daily was determined by subtracting the amount remaining from the original weight (10 g) of bait provided. All baits were weighed 24 h after removal from the cage to allow them to dry in case of urine contamination affecting bait weight. The percentage consumption of bait by weight was calculated.

### Reproductive physiology

A total of 47 males and 49 females were used for histological observations of the reproductive organs using animals fed on contraceptive bait for seven days. Animals were anaesthetized using diethyl ether and killed by cervical dislocation on day eight. On dissection, female and male reproductive organs were observed in situ to note any abnormalities, e.g. uterine oedema. The uterus, ovaries, testes, epididymis and seminal vesicles were removed and weighed. The epididymis of each male animal was dissected in a glass Petri dish containing 1 ml of 0.85% normal saline. A drop of the suspension was examined under magnification for sperm motility observations using an ordinary light microscope at magnification 20×. Another drop of the suspension was used to prepare a smear for sperm morphology analysis according to WHO standard protocols (WHO 2010). Smears were air dried and fixed with a solution of diethyl ether and ethanol (50:50) for 30 min. The smears were stained using 10% Giemsa for 30 min, washed with running tap water, dried and examined under oil immersion at magnification 100× to assess the sperm morphology. Two hundred sperm were observed per slide, and the number of sperm with abnormalities was recorded and expressed as percentage abnormal. In order to carry out sperm counts, the remaining epididymis samples were placed in glass test tubes and kept at 4 °C for 2 h in order to release the sperm. The samples were then diluted 1:10 by adding 9 ml of distilled water and placed in a modified Fuchs Rosenthal (B.S.748) chamber, following the WHO standard protocol to count sperm (WHO 2010).

### Effects on birth rate and litter size

A total of 216 animals (108 male and 108 female adult *M. natalensis*) were used to determine the potential effects of the contraceptives on pregnancy rate and litter size. Animals were provided with 10 g of contraceptive bait at three different concentrations (10, 50 and 100 ppm), while control animals were fed 10 g of plain bait. The bait was delivered for seven consecutive days, and water was provided ad libitum. Each concentration of the bait was provided to three replicates of male and female animals for each of the two fertility compounds and their combination. After the seven days of baiting, animals were paired for 10 days in four combinations. Each of the three treated females per group was paired with either a treated or untreated male, and each of the untreated females per group was paired with a treated or untreated male. Thereafter, females were retained and fed plain bait for 30 days for observation of pregnancy and litter size. The number of pups born in each litter produced by pregnant females was counted, weighed and compared with the control batch. Females that were not pregnant from all treatments including controls (untreated females paired with untreated males) were killed after 40 days from the start of the trial for uterus and ovary observation. The uteri and ovaries were dissected, weighed with all normal and abnormal features noted.

### Data analysis

Statistical analyses were performed using XLSTAT version 2015.1.03.16409 (Addinsoft, Paris, France). Comparisons of organ weights and pregnancy rates were made by an analysis of variance (ANOVA) with Duncan’s Multiple Range Test to separate the means at the 95% confidence interval. Bait uptake and body mass comparisons were made with a repeated measures (using daily measurements) ANOVA (least squares) followed by Duncan’s MRT.

## Results

### Bait acceptance and weight loss

The mean consumption of bait by rodents when individually grouped by sex, treatment and day was generally the same, with a few minor significant differences and general trends to be noted. (Table 1). Both male and female rodents fed quinestrol at 100 ppm consumed less bait on day 1 compared to the control group; however, all other treatment groups did not significantly vary from the control on a daily basis. Using daily measures of consumption in a repeated measures analysis of variance showed that there was no significant difference in the consumption rate between females (2.88 g/day) and males (3.03 g/day) (*F* = 0.389, *df* = 1, *P* = 0.533). However, consumption did vary by treatment with QE (2.24 g/day) and QE + LNG (2.20 g/day) showing reduced consumption compared to the control (4.0 g/day), with consumption in the LNG (3.74 g/day) treatment similar to the control (*F* = 21.856 *df* = 3, *P* < 0.0001). Increasing concentration also had an effect on consumption whereby bait consumption with the 10 ppm treatment (2.95 g/day) was not different from the untreated control (3.35 g/day) but where 50 ppm (2.76 g/day) and 100 ppm (2.75 g/day) showed significantly less bait consumption compared to the untreated bait (*F* = 4.600, *df* = 2, *P* = 0.010). Similarly on a daily basis, body mass of animals in different treatments did not significantly vary in comparison with the control group (Table 2). In a few instances, body mass dropped significantly for certain treatment groups. For example, males fed QE + LNG at 50 ppm had a significantly lower body mass from day 3 to 7, although no significant change was observed at the 10 and 100 ppm rates for males fed with the QE + LNG bait. A repeated measures ANOVA indicated that the sex (*F* = 0.202, *df* = 1, *P* = 0.653) of the animals and the concentration (*F* = 0.447, *df* = 2, *P* = 0.639) of the bait had no effect on body mass, whereas the treatment (*F* = 3.795, *df* = 3, *P* = 0.010) showed that the QE + LNG treatment had the lowest mean body mass (32.43 g), followed by QE (35.27 g), with the control (38.99 g) and LNG (38.92 g) treatments showing no difference from each other.

**Table 1:** Mean daily consumption of bait (g) by *Mastomys natalensis* containing different concentrations of quinestrol (QE), levonorgestrel (LNG) and quinestrol + levonorgestrel combination (QE + LNG) (*n* = 10, 5 males, 5 females)

Bait consumption (g/day)	Control	QE			LNG			QE + LNG		
		10 ppm	50 ppm	100 ppm	10 ppm	50 ppm	100 ppm	10 ppm	50 ppm	100 ppm
Day 1 female	5.34^ab^	2.96^abcdefgh^	3.40^abcdefgh^	1.14 ^h^	3.70^abcdefgh^	3.66^abcdefgh^	2.88^abcdefgh^	2.52^bcdefgh^	1.99^defgh^	4.08^abcdefgh^
Day 1 male	5.66^a^	2.68^abcdefgh^	1.84^efgh^	1.14^fgh^	4.54^abcdef^	3.68^abcdefgh^	4.60^abcde^	2.84^abcdefgh^	3.07^abcdefgh^	2.11^defgh^
Day 2 female	4.00^abcdefgh^	2.90^abcdefgh^	3.24^abcdefgh^	1.26^gh^	3.90^abcdefgh^	4.04^abcdefgh^	2.86^abcdefgh^	1.96^defgh^	1.44^fgh^	2.98^abcdefgh^
Day 2 male	5.06^abcd^	2.68^abcdefgh^	2.06^defgh^	1.26^abcdefgh^	5.28^abc^	4.22^abcdefgh^	3.4^abcdefgh^	2.48^bcdefgh^	2.55^abcdefgh^	2.16^cdefgh^
Day 3 female	2.84^abcdefgh^	3.06^abcdefgh^	3.02^abcdefgh^	1.74^efgh^	3.66^abcdefgh^	3.38^abcdefgh^	2.40^bcdefgh^	2.65^abcdefgh^	1.35^gh^	2.43^bcdefgh^
Day 3 male	2.74^abcdefgh^	2.80^abcdefgh^	2.26^bcdefgh^	1.74^efgh^	3.82^abcdefgh^	3.16^abcdefgh^	3.26^abcdefgh^	2.41^bcdefgh^	2.46^bcdefgh^	2.01^defgh^
Day 4 female	3.76^abcdefgh^	3.14^abcdefgh^	3.02^abcdefgh^	2.00^defgh^	4.14^abcdefgh^	3.40^abcdefgh^	2.26^bcdefgh^	2.27^bcdefgh^	2.22^bcdefgh^	2.41^bcdefgh^
Day 4 male	3.74^abcdefgh^	2.86^abcdefgh^	1.30^gh^	2.00^efgh^	3.86^abcdefgh^	3.44^abcdefgh^	3.36^abcdefgh^	2.17^cdefgh^	2.14^defgh^	2.30^bcdefgh^
Day 5 female	2.82^abcdefgh^	2.08^defgh^	3.06^abcdefgh^	1.58^efgh^	3.62^abcdefgh^	3.24^abcdefgh^	2.42^bcdefgh^	2.38^bcdefgh^	1.88^efgh^	2.07^defgh^
Day 5 male	3.62^abcdefgh^	2.72^abcdefgh^	1.34^gh^	1.58^efgh^	4.2^abcdefg^	3.46^abcdefgh^	3.56^abcdefgh^	2.02^defgh^	1.97^defgh^	1.79^efgh^
Day 6 female	2.66^abcdefgh^	2.52^bcdefgh^	2.20^cdefgh^	2.24^bcdefgh^	3.16^abcdefgh^	2.84^abcdefgh^	2.26^bcdefgh^	2.04^defgh^	1.95^defgh^	1.93^efgh^
Day 6 male	4.54^abcdef^	2.58^abcdefgh^	1.24^gh^	2.24^defgh^	3.76^abcdefgh^	2.36^bcdefgh^	3.28^abcdefgh^	1.87^efgh^	1.93^efgh^	2.34^bcdefgh^
Day 7 female	2.66^abcdefgh^	3.04^abcdefgh^	3.14^abcdefgh^	2.18^cdefgh^	3.50^abcdefgh^	3.18^abcdefgh^	2.58^abcdefgh^	2.23^bcdefgh^	2.26^bcdefgh^	2.04^defgh^
Day 7 male	3.88^abcdefgh^	2.12^defgh^	1.82^efgh^	2.18^defgh^	4.18^abcdefgh^	2.52^bcdefgh^	3.24^abcdefgh^	2.23^bcdefgh^	2.17^cdefgh^	1.73^efgh^
Female mean	3.44^abcdefgh^	2.81^abcdefgh^	3.01^abcdefgh^	1.73^defgh^	3.67^abcdefgh^	3.39^abcdefgh^	2.52^abcdefgh^	2.29^bcdefgh^	1.87^cdefgh^	2.56^bcdefgh^
Male mean	4.18^abcdefgh^	2.63^abcdefgh^	1.69^defgh^	1.97^defgh^	4.25^abcdefgh^	3.26^abcdefgh^	3.54^abcdefgh^	2.29^bcdefgh^	2.33^bcdefgh^	2.06^bcdefgh^

Repeated measures ANOVA (least squares) where means followed by different letters differ significantly at *P* < 0.05 (Duncan’s Multiple Range Test)

**Table 2:** Mean changes in animal body mass (g) of *Mastomys natalensis* when fed bait treated with quinestrol (QE), levonorgestrel (LNG) and quinestrol + levonorgestrel combination (QE + LNG) (*n* = 10, 5 males, 5 females)

Daily mean body mass (g)	Control	QE			LNG			QE + LNG		
		10 ppm	50 ppm	100 ppm	10 ppm	50 ppm	100 ppm	10 ppm	50 ppm	100 ppm
Day 0 female	34.20^abcdefgh^	32.18^abcdefgh^	37.06^abcdefgh^	40.38^abcdefgh^	41.46^abcdefgh^	41.06^abcdefgh^	38.78^abcdefgh^	32.38^abcdefgh^	34.39^abcdefgh^	33.56^abcdefgh^
Day 0 male	44.62^abcde^	34.44^abcdefgh^	37.22^abcdefgh^	40.42^abcdefgh^	43.68^abcdefg^	33.08^abcdefgh^	38.4^abcdefgh^	35.64^abcdefgh^	30.57^efgh^	35.23^abcdefgh^
Day 1 female	34.02^abcdefgh^	32.20^abcdefgh^	36.90^abcdefgh^	40.08^abcdefgh^	41.32^abcdefgh^	40.96^abcdefgh^	38.58^abcdefgh^	31.26^cdefgh^	32.60^abcdefgh^	33.85^abcdefgh^
Day 1 male	44.56^abcde^	34.12^abcdefgh^	36.78^abcdefgh^	40.06^abcdefgh^	43.52^abcdefg^	33.16^abcdefgh^	37.9^abcdefgh^	34.93^abcdefgh^	30.66^efgh^	34.15^abcdefgh^
Day 2 female	33.86^abcdefgh^	31.90^abcdefgh^	36.78^abcdefgh^	38.50^abcdefgh^	41.24^abcdefgh^	40.64^abcdefgh^	37.96^abcdefgh^	31.17^cdefgh^	32.69^abcdefgh^	33.74^abcdefgh^
Day 2 male	44.84^abcde^	34.08^abcdefgh^	35.78^abcdefgh^	38.66^abcdefgh^	43.72^abcdefg^	32.58^abcdefgh^	38.04^abcdefgh^	34.48^abcdefgh^	30.33^efgh^	33.93^abcdefgh^
Day 3 female	33.82^abcdefgh^	32.20^abcdefgh^	36.50^abcdefgh^	38.00^abcdefgh^	40.94^abcdefgh^	40.22^abcdefgh^	37.74^abcdefgh^	31.25^cdefgh^	31.81^abcdefgh^	33.55^abcdefgh^
Day 3 male	44.80^abcde^	33.88^abcdefgh^	35.04^abcdefgh^	38.00^abcdefgh^	43.52^abcdefg^	32.88^abcdefgh^	37.40^abcdefgh^	34.19^abcdefgh^	29.79^fgh^	33.64^abcdefgh^
Day 4 female	34.32^abcdefgh^	31.74^abcdefgh^	35.92^abcdefgh^	37.80^abcdefgh^	40.86^abcdefgh^	39.94^abcdefgh^	37.28^abcdefgh^	31.01^defgh^	31.54^bcdefgh^	33.08^abcdefgh^
Day 4 male	45.70^abc^	33.32^abcdefgh^	34.40^abcdefgh^	37.40^abcdefgh^	44.0^abcdef^	32.94^abcdefgh^	37.70^abcdefgh^	33.37^abcdefgh^	29.21^gh^	33.15^abcdefgh^
Day 5 female	34.40^abcdefgh^	30.62^efgh^	34.68^abcdefgh^	36.40^abcdefgh^	40.86^abcdefgh^	39.54^abcdefgh^	36.04^abcdefgh^	30.69^efgh^	31.39^bcdefgh^	32.71^abcdefgh^
Day 5 male	46.24^a^	31.86^abcdefgh^	32.98^abcdefgh^	35.10^abcdefgh^	43.46^abcdefg^	32.70^abcdefgh^	36.98^abcdefgh^	33.29^abcdefgh^	28.64^h^	32.65^abcdefgh^
Day 6 female	33.44^abcdefgh^	30.56^efgh^	34.40^abcdefgh^	35.72^abcdefgh^	40.12^abcdefgh^	39.42^abcdefgh^	35.80^abcdefgh^	29.87^fgh^	30.91^efgh^	32.69^abcdefgh^
Day 6 male	45.54^abcd^	31.82^abcdefgh^	32.60^abcdefgh^	34.58^abcdefgh^	43.52^abcdefg^	32.50^abcdefgh^	37.00^abcdefgh^	32.35^abcdefgh^	28.20^h^	32.38^abcdefgh^
Day 7 female	33.92^abcdefgh^	30.62^efgh^	34.30^abcdefgh^	36.68^abcdefgh^	39.94^abcdefgh^	39.28^abcdefgh^	35.76^abcdefgh^	29.94^fgh^	31.01^defgh^	32.14^abcdefgh^
Day 7 male	45.90^ab^	31.60^bcdefgh^	32.30^abcdefgh^	34.34^abcdefgh^	43.96^abcdef^	32.52^abcdefgh^	37.16^abcdefgh^	32.76^abcdefgh^	28.11^h^	32.03^abcdefgh^
Mean female	34.00^abcdefgh^	31.50^bcdefgh^	35.82^abcdefgh^	37.95^abcdefgh^	40.84^abcdefgh^	40.13^abcdefgh^	37.24^abcdefgh^	30.95^abcdefgh^	32.04^abcdefgh^	33.16^abcdefgh^
Mean male	45.28^abcde^	33.14^abcdefgh^	34.64^abcdefgh^	37.32^abcdefgh^	43.68^abcdef^	32.80^abcdefgh^	37.58^abcdefgh^	33.88^abcdefgh^	29.44^efgh^	33.40^abcdefgh^

Repeated measures ANOVA (least squares) where means followed by different letters differ significantly at *P* < 0.05 (Duncan’s Multiple Range Test)

### Reproductive physiology

**Fig. 1:**
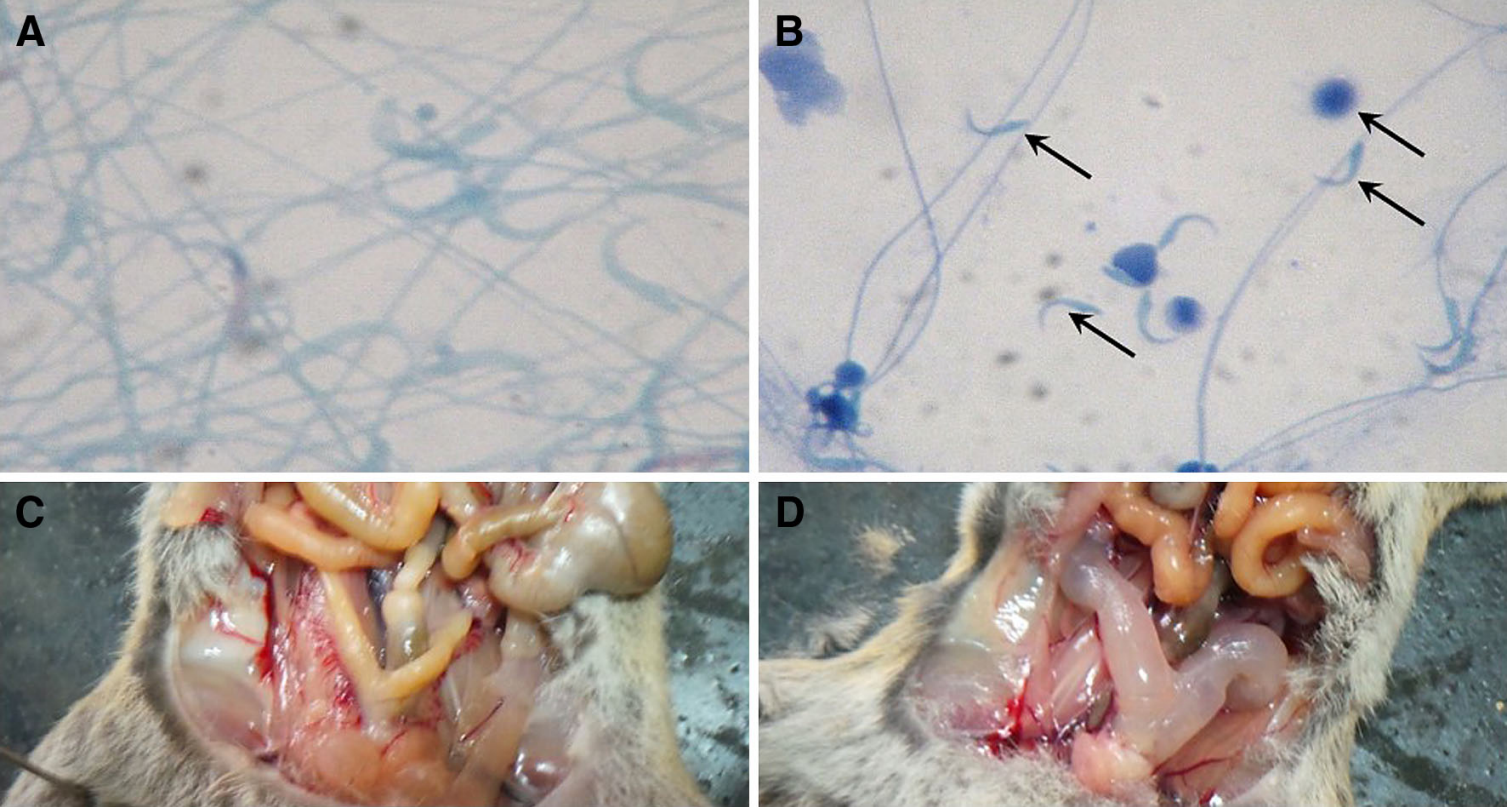
Physiological effect on male and female *Mastomys natalensis* when feeding on bait treated with fertility compounds. **a** Normal sperm when males fed on untreated bait (400 × magnification); **b** Sperm morphology in male *M. natalensis* when fed on quinestrol (QE), or quinestrol + levonorgestrel combination (QE + LNG) bait showing, at arrows, sperm with head and tail separated (400 × magnification); **c** Normal uterus in females fed on untreated bait. **d** Uterine oedema typically observed in female *M. natalensis* when fed on QE or QE + LNG treated bait at 8 and 40 days from trial start

On dissection, all males and females were considered to be sexually mature; with all males the testes were fully descended, and with females all uteri were fully vascularized. There was no difference in uterine or ovarian weights (*P* > 0.05) or structure except for a marked uterine oedema in animals feeding on bait treated with quinestrol and quinestrol + levonorgestrel at all concentrations, both for females killed at 8 and 40 days from trial start (Fig. 1). Effects of hormone treatments on testis weight, epididymis weight and sperm count were significant. On its own, levonorgestrel did not reduce the weight of the testes and epididymis but did have an effect on the seminal vesicle weight (Table 3), whereas quinestrol did not have a significant effect on the weight of the epididymis but did lower the weight of the testes and seminal vesicle. The effects of the two hormones together suggest that most of the organ weight reduction can be attributed to quinestrol. However, the QE + LNG combination does appear to have advantages in terms of reducing sperm motility below that achieved by either compound on its own (Table 3). The weight of seminal vesicles of all animals feeding on bait treated with contraceptive treated was lower (*F* = 11.55, *df* = 9, *P* < 0.0001) than that of the control group (Table 3). The lowest weight of seminal vesicles was observed in animals feeding on bait treated with QE and QE + LNG. Sperm concentration and motility decreased significantly (*F* = 13.18, *df* = 9, *P* < 0.0001, and *F* = 25.34, *df* = 9, *P* < 0.0001) in treated animals (Table 3). A significant (*F* = 22.10, *df* = 8, *P* < 0.0001) increase of abnormal morphology of sperm occurred in animals feeding on bait treated with QE and QE + LNG but not with LNG alone compared to the control group (Fig. 1).

**Table 3:** Mean (± SE) effects of quinestrol (QE), levonorgestrel (LNG), and quinestrol + levonorgestrel (QE + LNG) on male reproductive parameters. Values were obtained on day 8 of the trial (*n* = 5)

Parameter	Control	QE			LNG			QE + LNG		
		10 ppm	50 ppm	100 ppm	10 ppm	50 ppm	100 ppm	10 ppm	50 ppm	100 ppm
Testes weight (mg)	840 ± 0.06^a^	530 ± 0.09^bc^	380 ± 0.09^c^	370 ± 0.09^c^	740 ± 0.09^ab^	740 ± 0.09^ab^	860 ± 0.09^a^	440 ± 0.11^c^	380 ± 0.09^c^	390 ± 0.11^c^
Epididymis weight (mg)	770 ± 0.06^a^	600 ± 0.12^ab^	420 ± 0.11^ab^	430 ± 0.12^ab^	530 ± 0.11^ab^	640 ± 0.11^ab^	720 ± 0.11^ab^	390 ± 0.12^b^	510 ± 0.11^ab^	430 ± 0.12^ab^
Seminal vesicle weight (mg)	370 ± 0.02^a^	50 ± 0.05^d^	30 ± 0.04^d^	50 ± 0.04^d^	210 ± 0.04^bc^	130 ± 0.04^bcd^	260 ± 0.04^ab^	80 ± 0.05^d^	120 ± 0.04 ^cd^	80 ± 0.05^d^
Sperm count	2198.4 ± 151.6^a^	286.3 ± 96.9^b^	181.3 ± 62.6^b^	12.8 ± 7.64^d^	719.0 ± 262.6^b^	691.0 ± 162.6^b^	586.8 ± 122.6^b^	234.1 ± 96.9^b^	22.0 ± 12.6^c^	31.3 ± 6.9^c^
Sperm motility (%)	86.0 ± 4.41^a^	32.5 ± 8.63 ^cd^	12.8 ± 7.64^de^	15.0 ± 7.64^de^	60.0 ± 7.64^b^	32.0 ± 7.64 ^cd^	50.0 ± 7.64 ^cd^	1.2 ± 0.3^e^	1.4 ± 0.6^e^	1.2 ± 0.6^e^
Abnormal sperm morphology (%)	16.0 ± 3.28^d^	77.19 ± 6.45^ab^	86.01 ± 7.53^ab^	66.93 ± 6.43^b^	26.0 ± 5.69 ^cd^	24.78 ± 6.43 ^cd^	30.0 ± 5.69 ^cd^	50.63 ± 7.53^c^	95.24 ± 13.44^a^	*

ANOVA where means followed by different letters differ significantly at *P* < 0.05 (Duncan’s Multiple Range Test)

* No sperm produced

### Effects on birth rate and litter size

Time to delivery was similar for all treatments (23 ± 3 days). For QE and QE + LNG treatments, pregnancy and litter size were significantly reduced (*F* = 20.17, *df* = 3, *P* < 0.0001; *F* = 16.22, *df* = 3, *P* < 0.0001, respectively) in animals fed on treated bait that were paired with those of either sex fed on untreated or treated bait compared to the control (untreated males with untreated females) (Table 4). There were no pregnancies in females paired with treated males or when both sexes were fed on treated bait. When both sexes were left untreated, the average litter size was four pups.

**Table 4:** Mean pregnancy and litter size per pregnancy with respect to animals fed with quinestrol (QE), levonorgestrel (LNG) and quinestrol + levonorgestrel (QE + LNG) treatments at three concentrations (10, 50, 100 ppm) when put into mated pairs (*UNFUNM-*untreated female paired with untreated male, *TFUNM-*treated female paired with untreated male, *UNFTM-*untreated female paired with treated male, *TFTM-*treated female paired with treated male)

Activity	*n*	Mean pregnant females	Mean litter size/pregnancy
Treatment			
LNG	36	0.36 ± 0.07^a^	2.4 ± 0.6^a^
QE + LNG	36	0.2 ± 0.06^b^	1.4 ± 0.5^b^
QE	36	0.1 ± 0.03^b^	0.4 ± 0.3^c^
Concentration (ppm)			
100	36	0.2 ± 0.07^b^	1.9 ± 0.6^b^
50	36	0.1 ± 0.05^b^	1.2 ± 0.5^b^
10	36	0.2 ± 0.06^b^	1.1 ± 0.4^b^
Male–female pairs			
UNFUNM	27	0.4 ± 0.09^a^	3.7 ± 0.8^a^
TFUNM	27	0.2 ± 0.08^b^	1.6 ± 0.6^b^
UNFTM	27	0.0 ± 0.03^bc^	0.3 ± 0.3^bc^
TFTM	27	0.0 ± 0^c^	0.0 ± 0^c^
TFTM 10 ppm	9	0.0 ± 0^c^	0.0 ± 0^c^
TFUNM 10 ppm	9	0.2 ± 0.1^bc^	0.9 ± 0.6^b^
UNFUNM 10 ppm	9	0.4 ± 0.2^a^	3.7 ± 1.4^a^
UNFTM 10 ppm	9	0.0 ± 0^c^	0.0 ± 0^c^
TFTM 50 ppm	9	0.0 ± 0^c^	0.0 ± 0^c^
TFUNM 50 ppm	9	0.2 ± 0.1^bc^	1.9 ± 1.2^ab^
UNFUNM 50 ppm	9	0.3 ± 0.2^ab^	2.9 ± 1.4^ab^
UNFTM 50 ppm	9	0.0 ± 0^c^	0.0 ± 0^c^
TFTM 100 ppm	9	0.0 ± 0^c^	0.0 ± 0^c^
TFUNM 100 ppm	9	0.2 ± 0.1^bc^	2.1 ± 1.4^ab^
UNFUNM 100 ppm	9	0.6 ± 0.2^c^	4.6 ± 1.5^a^
UNFTM 100 ppm	9	0.1 ± 0.1^b^	0.9 ± 0.8^b^

ANOVA where means followed by different letters differ significantly at *P* < 0.05 (Duncan’s Multiple Range Test)

## Discussion

Rodent bait containing quinestrol and/or levonorgestrel was consumed by *M. natalensis* at a lower rate when compared to untreated bait. These findings are in contrast with other studies that showed no significant differences in bait consumption were associated with the concentration of fertility compounds in baits fed to other species of rodent. For example, Liu et al. (2012) showed that there were no significant differences caused by the concentration of quinestrol and levonorgestrel in bait fed to plateau pikas. Wang et al. (2011) observed no significant differences between treated and control groups on bait uptake by Brant’s voles (*Lasiopodomys brandtii*). The loss of weight in animals feeding on bait treated with fertility compounds could be attributed to low feed intake. Similar results have been reported in Mongolian gerbils (*Meriones unguiculatus*) (Lv and Shi 2011, 2012) after consuming bait containing quinestrol but not after consuming levonorgestrel. However, Liu et al. (2013) reported that quinestrol had no effects on the body weight of *Rattus nitidus* of either sex over seven days of treatment. In the current study, animals treated with quinestrol and levonorgestrel alone experienced much lower weight loss than those treated with the quinestrol + levonorgestrel combination. Although we cannot entirely discount the possibility that observed weight loss in some treatments affected reproductive success in our trial, on dissection all male testes were fully descended and all female uteri were considered to be sexually mature by level of vascularization. As female *M. natalensis* are known to successfully reproduce at body mass levels as low as 27 g (Coetzee 1965), we argue that all animals were sexually mature. Furthermore, pairing studies were performed with animals fed on plain bait, which may mitigate any physiological effects of weight loss experienced during the prior 7 days feeding on treated bait. More research using feeding choice tests and histological examination of ovarian and uterine tissues could help separate nutritional effects from hormonal effects.

In the current study, quinestrol and levonorgestrel had some effects on the reproductive status of both male and female *M*. *natalensis*. The results demonstrated that after 7 days of bait consumption, the weight of male reproductive organs decreased, with some differences depending on treatment. On its own, levonorgestrel did not reduce the weight of the testes and epididymis but did have an effect on the seminal vesicle weight, whereas quinestrol did not have a significant effect on the weight of the epididymis but did lower the weight of the testes and seminal vesicle. The effects of the two hormones together suggest most of the organ weight reduction can be attributed to quinestrol. However, the QE + LNG combination does appear to have advantages in terms of reducing sperm motility below that achieved by either compound on its own. This demonstrates that quinestrol and levonorgestrel have anti-fertility effects on male *M*. *natalensis*. Among the treatments, quinestrol and quinestrol + levonorgestrel at 10, 50 and 100 ppm were the most effective in reducing spermatogenesis, which has also been observed in other rodent species (O’Donnell et al. 2001). According to Li et al. (2014), quinestrol reduces semen quality and this may be caused by affecting processes such as sperm maturity in the epididymis and seminal vesicle secretion rates (Gonzales 2001). Various studies (Wang et al. 2011; Liu et al. 2012, 2014; Zhang 2015) have demonstrated that the concentration of these fertility compounds have significant reproductive effects on male rodents, including the greater long-tailed hamster (*Tscherskia triton*), Brandt’s vole (*Lasiopodomys brandtii*), the plateau pika (*Ochotona curzoniae*) and laboratory mouse (*Mus musculus*). Although in the current study, we did not determine the effects the fertility compounds have on subsequent offspring of animals which consumed treated bait, other studies have demonstrated that offspring of mothers treated with quinestrol were infertile whereas all male and female offspring from levonorgestrel-treated mothers were fertile (Lv et al. 2012).

Our findings show that the consumption of either quinestrol alone or quinestrol + levonorgestrel at the lowest concentration of 10 ppm for 7 days was sufficient to induce infertility in male *M. natalensis* for at least 10 days post-treatment. Although some sperm are still produced at this treatment dose, the observed reduction in sperm number and sperm quality is accepted as male infertility (WHO 2010). This has been observed in other rodent species including the greater long-tailed hamster (Zhang et al. 2005) fed on bait containing quinestrol and levonorgestrel at 10 and 30 ppm. Zhao et al. (2007) also reported that a dosage of quinestrol of 0.35 mg/kg body weight for male voles is effective to control this species in the field. According to Lv and Shi (2011), multiple dosages of 10 ppm of quinestrol + levonorgestrel delivered at one week intervals showed higher anti-fertility effects on female Mongolian gerbils than a single dosage treatment.

In female *M*. *natalensis* treated with quinestrol, uterine oedema was observed both at 8 days and 40 days, i.e. up to 33 days post-treatment. This has been attributed to abnormal amounts of oestrogen and progesterone which has been observed to lead to structural changes of the uterus in other species of rodents (Lv and Shi 2011). However, such changes to the uterus are not found in all rodents treated with these fertility compounds. Zhao et al. (2007) reported no significant differences in ovaries and uteri of Brandt’s voles treated with quinestrol, levonorgestrel and their combination, whereas Liu et al. (2013) found reduced weight of ovaries but not uteri of *Rattus nitidus* treated with quinestrol. Lv and Shi (2011) also reported increased gonadosomatic indices of uteri and reduced gonadosomatic indices of ovaries after quinestrol treatment in Mongolian gerbils. Lv and Shi (2012) reported that quinestrol increases the weight of the uterus, while the ovary weight remained unchanged in young females borne from quinestrol-treated mothers, but not in levonorgestrel-treated mothers of Mongolian gerbils. These inconsistent findings in female reproductive organs might be caused by interspecies differences of oestrogen and progesterone sensitivity in the reproductive organs of different species of rodents as exemplified by Lv and Shi (2011). Furthermore, Huo et al. (2006) reported changes in uterine structure in more than 50% of female Mongolian gerbils treated with 1 mg/kg (1 ppm) body weight of quinestrol/levonorgestrel combination, and that the uteri were severely disrupted by higher dosages.

In *M*. *natalensis* the fertility compounds affected both pregnancy and litter size, with the most effective compound in reducing the number of pregnancies being quinestrol. Fertility control was effective when both sexes had been fed on treated bait although there were no significant differences between untreated females paired with treated males and treated females paired with treated males. If both sexes were treated with the different concentrations of each compound no pregnancies were observed, whereas when both sexes were left untreated the average litter size was four pups. In our pairing study, sample size was relatively low (*n* = 3) and further replication will help confirm these observed effects. Evidence from other studies on other rodent species does support our results. Field studies in China indicated that reduced pregnancy and litter size per pregnancy was reduced by 60% in the Campbell’s dwarf hamster (*Phodopus campbelli*) treated with 0.01% quinestrol + levonorgestrel combination (Wan et al. 2006). Studies on other species of rodents have shown that there was reduced pregnancy rate and litter size in female Brandt’s vole paired with treated males (Wang et al. 2011). Lv and Shi (2012) found reduced litter size of females from quinestrol-treated mothers in contrast to levonorgestrel-treated mothers. Liang et al. (2006) confirmed the effectiveness of quinestrol + levonorgestrel combination in reducing fertility in male and female Mongolian gerbils.

Oestrogenic compounds routinely used in agriculture, livestock production and for human contraception are leading to environmental contamination. Thus, registration authorities in some parts of the world are sceptical about using hormones and particularly oestrogenic compounds that may contribute to environmental contamination and adverse effects on non-target species including humans. However, not all endocrine disruptors are the same, and some research indicates that quinestrol and levonorgestrel decompose quickly under field conditions, with half-lives of 5–16 days in soil and less than 3 days in water (Tang et al. 2012a, b; Zhang et al. 2014). Considering the well-known risks of many rodenticides in the environment and increased restrictions on their use, the registration of fertility control products for rodent pest management should be considered. Contraceptive baits would not be appropriate in all situations and unlikely to be acceptable in many household and urban situations; however, limiting rodent reproduction in areas where population irruptions occur could be highly sustainable with minimal non-target effects.

This study is the first to report on the effects of anti-fertility compounds on an outbreaking rodent pest species found throughout sub-Saharan Africa. The anti-fertility effects of synthetic steroid hormones (quinestrol and levonorgestrel) in rats have shown potential to control other species of rodents (Lv and Shi 2011; Liu et al. 2013). From our preliminary studies in the laboratory, the effects on fertility of *M*. *natalensis* seem very promising for field experimentation to reduce populations of this species which can have litter sizes of 3–17 pups and often reach >200 animals per hectare in crop fields (Coetzee 1965; Makundi and Massawe 2011). The next steps in this research would be to carry out bait feeding choice tests and field studies comparing the use of baits with rodenticide and anti-fertility compounds on the population dynamics of *M. natalensis* that also monitor agricultural crop damage levels.

## Author contributions

SRB, RHM, ZZ, AWM, HJL and ML conceived and designed the experiments; GM, AW and RHM performed the experiments; GM, AWM and SRB analysed the data; and AWM, RHM, SRB, ZZ, GM, ML and HJL wrote the paper.
